# Role of Radiosurgery and Stereotactic Ablative Radiotherapy for Oligometastatic Non-Oncogene Addicted NSCLC

**DOI:** 10.3390/cancers14061465

**Published:** 2022-03-12

**Authors:** Serena Badellino, Mario Levis, Erica Maria Cuffini, Marzia Cerrato, Erika Orlandi, Ilaria Chiovatero, Arianna Aprile, Alessio Gastino, Chiara Cavallin, Giuseppe Carlo Iorio, Ramona Parise, Cristina Mantovani, Umberto Ricardi

**Affiliations:** Department of Oncology, University of Torino, 10125 Torino, Italy; serena.badellino@tiscali.it (S.B.); erica.cuffini@gmail.com (E.M.C.); mrz.cerrato@gmail.com (M.C.); dottoressaerika@gmail.com (E.O.); ilakiowa@hotmail.it (I.C.); arianna.aprile@edu.unito.it (A.A.); alessio.gastino@gmail.com (A.G.); chia.cavallin@gmail.com (C.C.); giuseppecarlo.iorio@libero.it (G.C.I.); pariseramona@gmail.com (R.P.); cristina.mantovani@ymail.com (C.M.); umberto.ricardi@unito.it (U.R.)

**Keywords:** oligometastatic disease, NSCLC, SABR, SRS, ablative treatment, non-oncogene addicted NSCLC

## Abstract

**Simple Summary:**

The definition and management of oligometastatic NSCLC have been incorporated into current guidelines on lung cancer, supporting the use of a definitive treatment with curative intent, including the focal ablation of all sites of oligometastatic involvement. Clinical evidence highlighting the use of local ablative treatment (LAT), alone or in combination with systemic therapies, demonstrated significant benefit in local control and progression-free survival, especially in “oncogene-addicted” patients, who benefit the most from the local treatment of oligoprogressive or oligorecurrent sites to restore the overall sensitivity of the metastatic disease to target therapies. On the other hand, only a few studies, with limited numbers, focused specifically on “non-oncogene” addicted patients. The aim of this study is to assess the role of LAT, referred to stereotactic ablative radiotherapy, and to report clinical outcomes of the largest retrospective series, to date, of patients with oligometastatic EGFR/ALK/ROS1 wild type NSCLC.

**Abstract:**

Local ablative therapy (LAT), intended as stereotactic ablative radiotherapy or stereotactic radiosurgery, is a well-recognized effective treatment for selected patients with oligometastatic NSCLC. Current clinical evidence supports LAT alone or in combination with systemic therapies. Our retrospective mono-institutional study aims to assess the role of LAT with a peculiar focus on the largest series of non-oncogene addicted oligometastatic NSCLC patients to date. We included in this analysis all patients with the mentioned disease characteristics who underwent LAT for intracranial and/or extracranial metastases between 2011 and 2020. The main endpoints were local control (LC), progression free survival (PFS) and overall survival (OS) in the whole population and after stratification for prognostic factors. We identified a series of 245 consecutive patients (314 lesions), included in this analysis (median age 69 years). In 77% of patients, a single metastasis was treated with LAT and intracranial involvement was the most frequent indication (53% of patients) in our series. The overall response rate (ORR) after LAT was 95%. In case of disease progression, 66 patients underwent new local treatments with curative intent. With a median follow-up of 18 months, median PFS was 13 months (1-year PFS 50%) and median OS was 32 months (1-year OS 75%). The median LC was not reached (1-year LC 89%). The presence of brain metastases was the only factor that negatively affected all clinical endpoints, with a 1-year LC, PFS and OS of 82%, 29% and 62% respectively, compared to 95%, 73% and 91%, respectively, for patients without BMs (*p* < 0.001 for each endpoint). At the multivariate analysis, mediastinal nodal involvement at baseline (*p* = 0.049), ECOG PS = 1 (*p* = 0.011), intracranial disease involvement (*p* = 0.001), administration of chemotherapy in combination with LAT (*p* = 0.020), and no delivery of further local treatment for progression or delivery of focal treatment for intracranial progression (*p* < 0.001) were related to a poorer OS. In our retrospective series, which is to our knowledge the largest to date, LAT showed encouraging results and confirmed the safety and effectiveness of focal treatments in non-oncogene addicted oligometastatic NSCLC patients.

## 1. Introduction

Non-small cell lung cancer (NSCLC) is the leading cause of cancer death [1,2]. More than 50% of NSCLC patients present with stage IV disease at diagnosis or develop distant metastases after primary treatments [1]. Prognosis for these patients remains severe (5-year OS < 10%) [2]. In the last three decades a new category of metastatic NSCLC patients, as a sort of transitional state between locoregional disease and widespread systemic disease, has been identified, with a limited number of secondary lesions (usually from one to five) involving a limited number of organs (no more than three): this condition is referred to as oligometastatic disease (OMD) [3,4,5]. The prevalence of OMD among NSCLC patients has been estimated to range between 20% and 50%, depending on the different possibilities in terms of oncological scenario. The identification of molecular biomarkers (e.g., alterations in EGFR, ALK, ROS1 genes) that are druggable with targeted systemic therapies and the development of immune checkpoint inhibitors (ICIs) have modified the management and the prognosis of NSCLC, increasing the percentage of stage IV patients with an OMD and consequently improving the outcome [6,7].

The definition and management of oligometastatic NSCLC have been incorporated into current guidelines on lung cancer, supporting the use of a definitive treatment with curative intent directed to primary disease and including focal ablation of all sites of oligometastatic involvement [8,9,10,11]. In the pre-immunotherapy era, prospective phase 2 randomized trials have shown an increase in progression free survival (PFS) and overall survival (OS) for oligometastatic patients receiving radiotherapy (RT) as local ablative treatment (LAT) in combination with systemic therapy [12,13,14]. In recent years, there has been a growing interest in the potentially synergistic effect of LAT and novel systemic therapies (immunotherapy alone or in combination with chemotherapy) [15,16], but limited data are available and more robust evidence is expected from the ongoing trials (SARON and LONESTAR trials).

To date, many published reports include mainly “oncogene-addicted” patients, who benefit the most from the local treatment of oligoprogressive (OP) or oligorecurrent (OR) sites to restore the overall sensitivity of the metastatic disease to target therapies. On the other hand, only a few studies, with limited numbers, explicitly focused on “non-oncogene” addicted patients. In this paper, we present the results of LAT on the largest retrospective series of EGFR/ALK/ROS1 wild type oligometastatic NSCLC patients treated at our institution.

## 2. Materials and Methods

### 2.1. Study Design, Patient Population

In this retrospective mono-institutional study, we reviewed the medical charts of all patients treated with LAT for oligo-metastatic (OM), OR or OP non-oncogene addicted NSCLC at the Radiation Oncology Unit of the Department of Oncology of the University of Turin, between January 2011 and December 2020. We included a cohort of unselected and consecutive patients who received one or more of the following LATs: extracranial Stereotactic Ablative Radiotherapy (SABR) or brain Stereotactic Radiosurgery (SRS) or (hypo-)fractionated stereotactic RT (SRT), even with concomitant systemic therapy. The inclusion criteria were as follows: age ≥ 18 years; histologic diagnosis of NSCLC; no proven alterations in EGFR, ALK and ROS1 genes; stage IV synchronous OMD or metachronous OR/OP disease (ORD/OPD) according to the ESTRO-ASTRO consensus (OMD defined as 1–5 metastatic lesions in patients naïve to previous systemic therapy for stage IV NSCLC; ORD and OPD defined as 1–5 metastatic lesions in patients relapsing or progressing during systemic therapy/local treatments for metastatic disease [4].

In the few cases without histological confirmation, a new and/or increasing lung nodule, with abnormal positron emission tomography (18 FDG PET) uptake was defined as a secondary lesion. The differential diagnosis between secondary lesion or a new primary lung tumor was performed considering the natural history of the disease and the time interval elapsing between the NSCLC diagnosis and the onset of the new lesion, with a temporal cut-off set at 2 years as in previous reports from our Institution [17]. The dedicated tumor board discussed all cases. Patients with prior non-ablative RT treatments (e.g., surgery) were not excluded if at least one metastasis had been treated with SABR/SRS/SRT during the study period. Staging before LAT included whole-body computed tomography (CT) scans and 18FDG-CT-PET; in addition, magnetic resonance imaging (MRI) was employed in patients with brain metastases (BMs). Each patient signed an informed consent form for RT treatments. Our Institutional Review Board authorized the study.

### 2.2. Outcomes/Endpoints

The primary endpoints of this study were PFS and OS in the whole population and in the population stratified by site of lesions. PFS was defined as the time from the date of LAT to the date of first progression, local or distant, or to the date of death, whichever occurred first, with patients not experiencing any event censored at the date of last follow up; OS was defined as the time from the date of local ablative stereotactic RT of oligometastases to the time of death, with living patients censored at the date of last follow-up. The secondary endpoint was local metastasis control (LC).

### 2.3. Treatment Details

The following data were collected for LAT: dates for start and end of radiotherapy, number and sites of the treated lesions, RT details (SRS/SRT or SABR, cumulative tumor dose, number of fractions and dose per fraction and PTV volume). Treatment planning and delivery were described in conformity with the requirements of ablative RT treatments and as presented in detail in previous reports from our institution [18,19].

For brain lesions, SRS was offered to treat small or unresectable BMs with a risk-adapted approach depending on tumor diameter [20,21]. With a prescription dose of 18–21 Gy, SRS was preferred for lesions < 2 cm, while surgery or SRT were preferred when treating lesions with a diameter > 2 cm. After surgical resection of BMs, focal SRT was largely adopted as adjuvant treatment, again with a risk adapted strategy (24–30 Gy in 3–5 fractions).

For lung lesions, tumor selection criteria for SABR were as follows: a maximum tumor diameter below 50 mm, adequate pulmonary function, Eastern Cooperative Oncology Group Performance Status (ECOG PS) < 2. The prescription dose (always at 80%-isodose) ranged from 26 to 60 Gy in 1–8 fractions, depending on tumour location, according to a ‘‘risk-adapted” fractionation schedule. Dose constraints for organs at risk were derived from the latest recommendations available in the literature [22,23].

The preferred schedule for extra-thoracic lesions was 30–35 Gy in 5 fractions.

### 2.4. Response Assessment and Follow-Up

According to our internal stereotactic RT protocol, all patients received a clinical examination to assess any side effects within 1 month after the focal treatment. Response to treatment was evaluated based on radiological restaging with brain-thorax-abdomen CT scan with intravenous contrast media and, in the case of BMs, with MR scan. Response rates were classified as complete response (CR), partial response (PR), stable disease (SD) and progressive disease (PD) according to RECIST criteria v. 1.1 [24]. CR, PR and SD were combined to define the overall response rate (ORR).

Patients were then prospectively followed up with diagnostic imaging every 3 months for the first 2 years and then every 6 months, in order to detect any relapse and to assess the occurrence and grading of any toxicity. 18FDG-CT-PET scanning was requested during the follow-up only in selected cases. Medical records were checked to assess any treatment complication during and/or after RT.

### 2.5. Statistical Analyses

Categorical variables of patients’ baseline were presented as percentages, while continuous variables were summarized with medians and ranges. Univariate and Multivariate analyses were performed by employing the Cox proportional hazards regression model, with the backward exclusion of non-significant variables. Hazard ratios (HR) and 95% confidence intervals (95% CI) were estimated. OS rates and PFS were calculated with the Kaplan–Meier method using the start date of LAT to the first OM site treated, while subgroups were compared using the log-rank test. Variables identified as survival factors from the final multivariate analysis were then used as stratification factors for Kaplan–Meier survival curves. LC, PFS and OS were also evaluated through stratification of the population by site of treatment. All statistical analyses were performed using SPSS for Windows version 26.0 (SPSS Inc., Chicago, IL, USA) and a *p*-value < 0.05 was considered statistically significant.

## 3. Results

### 3.1. Population Characteristics

A total of 245 patients met the inclusion criteria and were included in this analysis. There was a clear prevalence of male sex (71%) and the median age was 69 years (range 39–85). All patients had an ECOG PS < 2. Adenocarcinoma was diagnosed in 74% of patients, squamous cell carcinoma in 14%, with 12% of patients presenting other or unspecified NSCLC subtypes. PDL-1 expression was investigated in only 20% of patients. At the time of initial diagnosis, 65 patients (27%) presented with early-stage NSCLC, 106 (43%) had a locally advanced disease and 74 (30%) were metastatic. A locoregional nodal involvement (N+) at baseline was detected in 125 patients (51%) at the first diagnosis of NSCLC. At the time of the LAT, 53 patients (22%) had synchronous OMD, 165 patients (67%) had ORD and 27 had OPD. Most patients (154, 63%) had a single metastasis, with 63 participants (25%) presenting two metastatic lesions. Only 28 (12%) patients had 3 to 5 lesions, mostly with brain involvement. A single organ was involved in 89% of the analyzed population, while the remaining 11% (27 patients) had secondary lesions in 2 or more organs (with a maximum of 4 involved organs). Lung involvement was detected in 115 patients (47%), while BMs were found in 129 patients (53%); adrenal metastastic deposits were detected in 13 patients (5%), while 6 participants had bone metastases (2%). All patients’ baseline characteristics are summarized in Table 1.

### 3.2. Treatment Characteristics

At the time of first NSCLC diagnosis, 84 patients (35%) received standardized combined regimens (chemotherapy plus radiotherapy, surgery plus adjuvant therapy), 101 (41%) were treated with focal therapies (71 surgery, 30 SABR), while 55/245 were treated with systemic therapy alone. At OMD diagnosis, the primary tumor was controlled in 195 patients (80%). All patients received LAT for OMD (either SABR or SRS/SRT), and other alternative local therapies (e.g., surgery or radiofrequency) were administered in 5% of cases; of the 10 patients who underwent surgery, 8 were treated for BMs and received adjuvant SABR/SRS. Only 2 patients received SABR/SRS on more than one organ. A total of 314 lesions underwent LAT. Most patients (187, 77%) were treated for a single metastasis, 48 (19%) had two lesions and 10 (4%) had 3–4. Chemotherapy was combined with LAT within two months since focal therapy in 71 (29%) patients; in this group, 53 patients had a diagnosis of synchronous OMD, 7 had OPD and 11 ORD. All treatment characteristics are summarized in Table 2.

### 3.3. Clinical Outcomes

The response to LAT was very good, with an ORR of 95% and only 11 patients (5%) experiencing PD at the first restaging after treatment. Of these 11 patients, 7 had BMs and 6 had two or more lesions before treatment.

**Figure 1:**
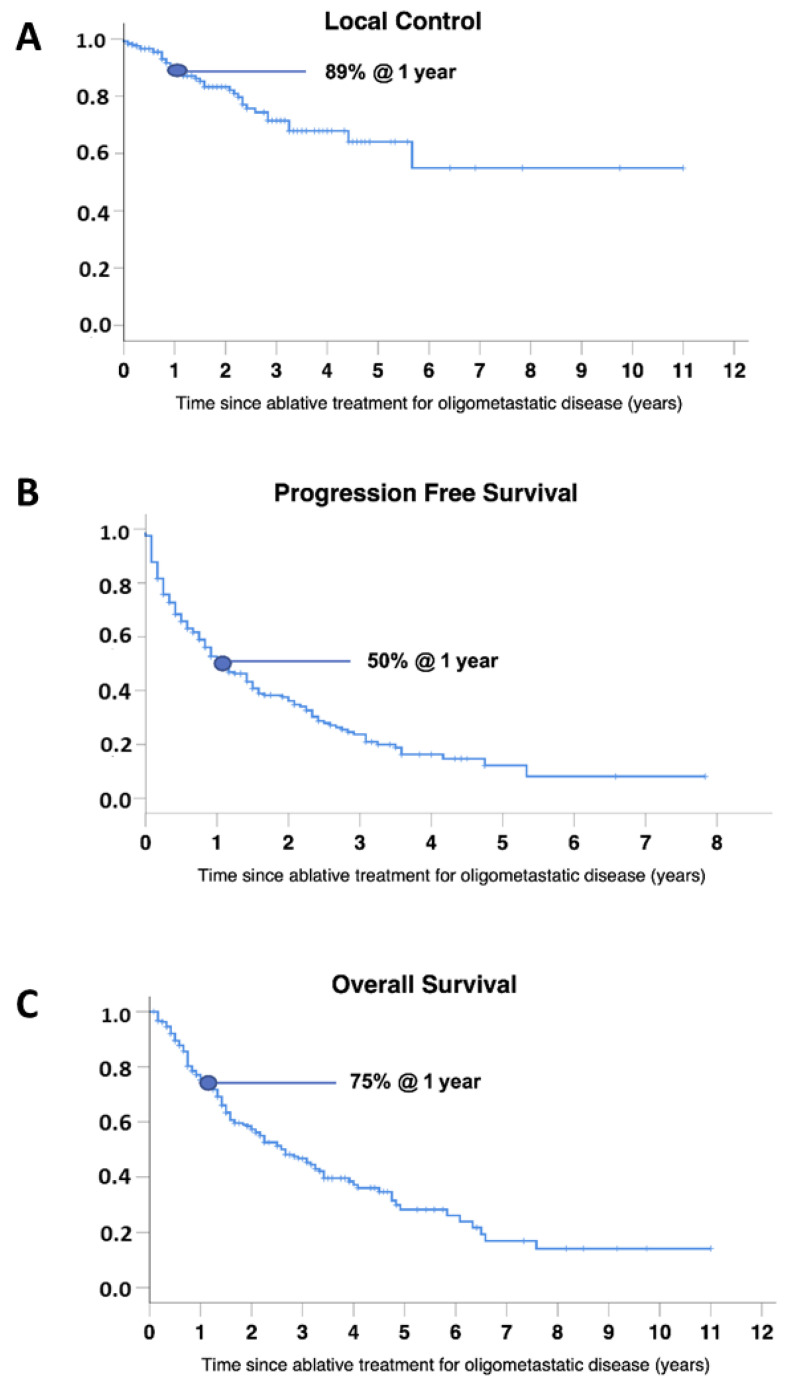
LC (**A**), PFS (**B**) and OS (**C**) in the overall population.

Median follow-up was 18 months in the overall population and 26 months in patients alive at the last follow-up (range 1–132 months). In the overall population: (1) the median LC was not reached, with 1-, 2- and 3-year LC of 89%, 83%, and 71%, respectively; (2) the median PFS was 13 months, with 1-, 2- and 3-year PFS of 50%, 36%, and 24%, respectively; (3) the median OS was 32 months, with 1-, 2- and 3-year OS of 75%, 57%, and 47%, respectively (Figure 1).

Ninety-nine patients (40%) received further local treatments for PD after the first LAT (62 to the brain and 37 to extracranial sites). In particular, 66 patients received new curative therapies such as surgery (7/66), SABR/SRS (53/66) and other combinations of focal treatments (e.g., whole brain radiotherapy + SRT boost) (6/66); palliative radiotherapy was administered in the other 33 patients. Of 164 patients with PD, 65 did not receive any local therapy.

At last follow-up, 131 patients (53%) were deceased.

At univariate analysis, treatment of >2 lesions (*p* = 0.005) and intracranial disease involvement (*p* = 0.001) were associated with poorer LC.

Factors predictive of reduced PFS at univariate analysis were: age (*p* = 0.009), N+ at baseline (*p* = 0.001), stage IV at diagnosis (*p* < 0.001), absence of response after primary treatment (*p* = 0.005) or after LAT for OMD (*p* < 0.001), ECOG PS = 1 (*p* = 0.001), synchronous OMD (*p* = 0.002), number of lesions at OM diagnosis ≥ 2 (*p* = 0.003), uncontrolled primary tumor (*p* = 0.002), administration of chemotherapy in combination with LAT (*p* < 0.001) and the presence of intracranial disease involvement (*p* < 0.001).

The following factors were found as predictors of lower OS at univariate analysis: N+ at baseline (*p* = 0.003), stage IV at diagnosis (*p* = 0.007), ECOG PS = 1 (*p* < 0.001), number of lesions at OMD (*p* = 0.007), uncontrolled primary tumor (*p* = 0,013), intracranial disease involvement (*p* < 0.001), administration of chemotherapy in combination with LAT (*p* = 0.003), intracranial PD after LAT and no delivery of further local treatment or delivery of focal treatment for new intracranial lesions in case of PD (*p* = 0.001).

A multivariate analysis was conducted for PFS and OS and included all variables affecting these outcomes at the univariate analysis. For PFS, administration of chemotherapy in combination with LAT (HR = 1.9, 95% CI = 1.2–2.7, *p* = 0.002), ECOG PS = 1 (HR = 1.9, 95% CI = 1.3–2.6, *p* < 0.001), intracranial disease involvement (HR = 2.4, 95% CI = 1.7–3.4, *p* < 0.001), and absence of response after LAT (HR = 4.4, 95% CI = 2.2–8.5, *p* < 0.001) had a negative impact. For OS, N+ at baseline (HR = 1.6, 95% CI = 1.1–2.4, *p* = 0.049), ECOG PS = 1 (HR = 1.7, 95% CI = 1.1–2.6, *p* = 0.011), intracranial disease involvement (HR = 2.3, 95% CI = 1.4–3.9, *p* = 0.001), administration of chemotherapy in combination with LAT (HR = 1.7, 95% CI = 1.1–2.6, *p* = 0.020) and no delivery of further local treatment or delivery of focal treatment for new intracranial lesions in case of PD (HR = 2.2, 95% CI = 1.6–3.1, *p* < 0.001) were related to a poorer outcome. Results from univariate and multivariate analyses for predictors of PFS or OS are detailed in Table 3.

**Figure 2:**
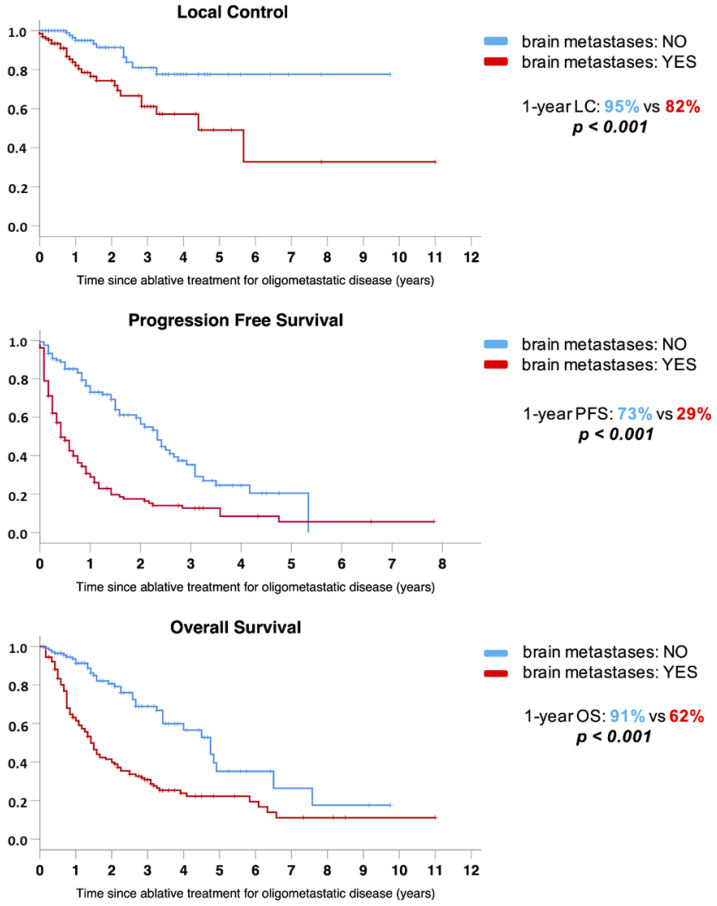
Outcomes stratified by the presence of intracranial disease involvement. Upper chart: LC; middle chart: PFS; lower chart: OS.

Overall, the presence of intracranial metastases at the time of OMD diagnosis was the only factor that negatively affected all clinical endpoints, as shown in Figure 2. One-, 2- and 3-year LC was 82%, 74% and 61% respectively for patients with BMs, while it was 95%, 91% and 81% for patients without BMs (*p* < 0.001). A significant difference was detected also in PFS, with 1-, 2- and 3-year rates of 29%, 18% and 13% versus 73%, 57% and 35%, respectively, for patients with and without BMs (*p* < 0.001). Lastly, median OS was significantly different for patients with and without BMs (17 vs. 57 months), with 1-, 2- and 3-year rates of 62%, 40% and 31% versus 91%, 81% and 69%, respectively (*p* < 0.001).

**Figure 3:**
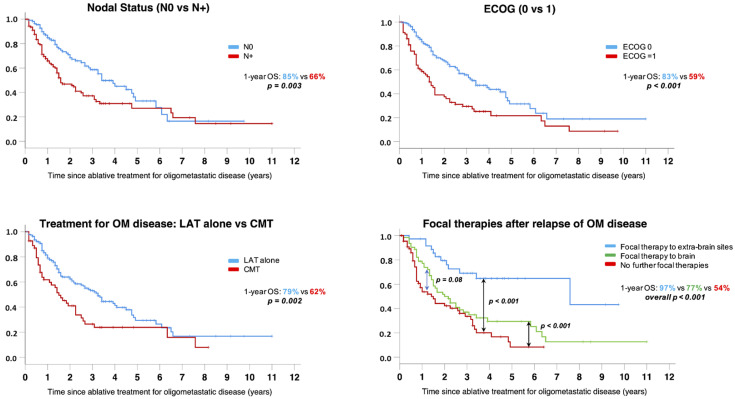
Subgroup analysis showing OS Kaplan–Meier curves stratified for the following variables: Nodal involvement (upper left chart), ECOG PS (upper right chart), addition of chemotherapy (CMT) to LAT (lower left chart) and the delivery of any further local treatment for relapse/progression after LAT (lower right chart).

Other prognostic factors affecting survival are shown in the Kaplan–Meier curves of Figure 3. A significant reduction in OS was seen: (1) in patients with N+ at baseline (1- and 2-year OS of 66% and 47%, respectively, for N+ patients compared to 85% and 68%, respectively, for N0 patients, *p* = 0.003); (2) in patients with ECOG PS = 1 (1- and 2-year OS of 59% and 37%, respectively, for patients with ECOG PS = 1 compared to 83% and 68%, respectively, for patients with ECOG PS = 0, *p* = 0.003); (3) in patients receiving chemotherapy in combination with LAT (1- and 2-year OS of 62% and 41%, respectively, for those receiving systemic therapy compared to 79% and 62%, respectively, for those receiving LAT alone, *p* = 0.002); (4) in patients relapsing after a first LAT who did not receive any further local therapy or who were treated with local therapy for brain progression, compared to patients receiving new local therapies for extracranial progression (1- and 2-year OS of 54% and 42%, respectively, for those not receiving further local therapies compared to 77% and 50%, respectively, for those receiving local therapy for brain relapse/progression and to 97% and 80%, respectively, for those receiving local therapy for extracranial relapse/progression, overall *p* < 0.001).

## 4. Discussion

Over the past 30 years oligometastatic NSCLC has been recognized as a unique clinical entity [3,4,5,11,25]. Several retrospective series and some prospective studies have demonstrated the efficacy and safety of aggressive approaches in selected patients with OMD and supported the use of metastasis-directed ablative radiotherapy alone, or in addition to a primary therapy to consolidate all sites of gross disease [26,27,28,29,30,31,32]. In addition, results from recent Phase II randomized trials have revealed a potential benefit for ablative RT in prolonging PFS [12] and OS [13,14].

Taken together, all these trials involved modest sample sizes with less than a hundred patients with lung cancer. Additionally, the prospective studies were performed in the pre-immunotherapy era, thereby excluding a treatment option that has radically shifted the management paradigm in NSCLC.

To our knowledge, this study reports the clinical outcomes of the largest retrospective series of oligometastatic non-oncogene addicted NSCLC patients treated with LAT. The admission to this study required the absence of activating mutations of the EGFR gene or ALK oncogene rearrangements, which made these patients ineligible for target therapy. With this premise, the median PFS of 13 months reported herein is consistent with previously published studies on oligometastatic NSCLC patients, with a 1-year risk of distant or local relapse of approximately 50% after the ablative treatment [13,14,27,29,30,33]. The median survival was 32 months, in accordance with current literature [13,27]. Our results are promising, since patients included in this series had no “druggable” molecular targets and were therefore deemed ineligible for treatment with tyrosine kinase inhibitors which are associated with high response rates and improved survival outcomes [7] in oligometastatic NSCLC. Moreover, only two patients were treated with ICIs and therefore our results are not influenced by the more recent introduction of immunotherapy in the clinical arena.

In this study, we detected the negative prognostic role for nodal disease involvement at baseline, suboptimal performance status and intracranial disease at the time of LAT, which reflect the results of previous reports [27,34].

In particular, the presence of BMs was the worst prognostic factor, affecting either LC, PFS and OS (median OS of 17 vs. 57 months for patients with and without BMs, respectively). The management of intracranial lesions still represents the most critical issue in NSCLC patients in general and especially in the OM setting, despite the increasing availability of modern technical solutions to deliver high doses of highly conformed and ablative RT [21]. Starting with the scarce efficacy of systemic agents because of the presence of the blood–brain barrier and with the necessity to deliver suboptimal RT doses (BED < 100 Gy constantly) compared to extracranial lesions (BED > 100 Gy recommended) [35] for the risk of radionecrosis [36], many factors influence the limited success of ablative RT on BMs in terms of LC (1-year rate of 82% compared to 95% for extracranial sites), which translates to lower OS rates.

Furthermore, almost one-third of patients received systemic therapy in combination with LAT. The poor prognosis of this subgroup (1-year OS of 62% vs. 79% for patients treated with LAT alone), may be caused primarily by the fact that most of these patients had a “de-novo” OM disease, which is a well-recognized negative prognostic factor compared to OPD/ORD [26,27,29], and secondarily by the fact that some areas of progression worthy of local consolidation therapy could not have been treated. This might result in isolated progression of untreated disease sites, which become persistent foci for future micrometastatic seeding [10,37] when systemic therapy alone has an unsatisfactory local effect in controlling the grossly visible disease. Ongoing randomized studies will clarify the role of ablative RT in combination with systemic therapy (NRG-LU002 and SARON trials).

We also detected a beneficial effect of further local treatments on new disease sites in those patients who progressed/relapsed after initial LAT. The benefit was observed mainly in patients with extracranial progression and to a minor extent in patients with intracranial progression, compared to patients who did not receive any local treatment (1-year OS 97% vs. 77% vs. 54%, respectively). These findings lead to considering the potential contribution of local therapies in case of further oligoprogression after the first focal treatment in OM disease.

Mimicking the information by oncogene-addicted NSCLC [38], it is possible for new local treatments to have the ability to restore overall chemo-sensitivity of the metastatic disease once relapsing lesions have been eradicated.

This study has a number of shortcomings. The main limitation is the mono-institutional and retrospective nature. Secondly, this analysis was conducted on a highly-selected population with favorable factors (good performance status, single metastases and single organ involvement in most patients) that may partially justify the encouraging results. For such reasons, an extrapolation of our findings for patients with even 3 to 5 metastases, or with multiple metastases in different organs, might not be appropriate. The lack of a standardized follow-up may have influenced the small number of OPD patients and incomplete data on several variables of interest such as PD-L1 expression rate. The latter point is highly relevant, in particular for future studies, given the discoveries on the role of immunotherapy in this setting and the resultant shift in the treatment paradigm. In our retrospective case series, conducted mostly on patients treated before the advent of immunotherapy in the daily routine, data regarding PD-L1 expression were available in only 20% of patients and only 2 received ICIs. Future prospective studies investigating the combination of (chemo)-immunotherapy plus SABR/SRS will be fundamental to demonstrate the possible efficacy and safety of this combination (NRGLU002 (NCT03137771), SARON (NCT02417662) and LONESTAR (NCT03391869)).

## 5. Conclusions

This large retrospective study showed encouraging results for LAT in non-oncogene addicted oligometastatic NSCLC. Patients in good clinical conditions, without intracranial metastases and without nodal involvement at baseline may have a better prognosis. Moreover, repeating local treatment on sites of further progression improves survival, especially for extracranial sites, and might be considered whenever possible. Our findings are in line with the literature and should be corroborated with further dedicated studies. Future prospective trials, investigating the combination of LATs with novel agents such as ICIs, are eagerly awaited to find new potential strategies able to further improve the outcome for these patients.

## Figures and Tables

**Table 1:** Patients baseline characteristics.

All Patients, *n* = 245 No. (%)	
Gender	
Male	174 (71)
Female	71 (29)
Age (years)	
<70	125 (51)
≥70	120 (49)
ECOG PS	
0	166 (68)
1	79 (32)
Histology	
Adenocarcinoma	181 (74)
Squamous Cell	34 (14)
Other	16 (6)
NSCLC Unknown/Not specified	14 (6)
PD-L1	
0%	21 (8)
>1%	15 (6)
>50%	14 (6)
Unknown	195 (80)
Stage at NSCLC diagnosis	
Early	65 (27)
Locally Advanced	106 (43)
Metastatic	74 (30)
N stage at NSCLC diagnosis	
0	114 (47)
1	39 (16)
2	66 (27)
3	20 (8)
Unknown	6 (2)
Type of OMD	
Synchronous	53 (22)
Oligorecurrent	165 (67)
Oligoprogressive	27 (11)
Lesion(s) at OMD diagnosis	
1	154 (63)
2	63 (25)
3	19 (8)
4	4 (2)
5	5 (2)
No. of involved organ(s) at OMD diagnosis	
1	218 (89)
2	25 (10)
3–4	2 (1)
Type of involved organ(s) at OMD diagnosis	
Lung	102 (41)
Brain	108 (44)
Bone	4 (2)
Adrenal	5 (2)
Multiple organs with brain ^a^	21 (9)
Multiple organs extra brain ^b^	5 (2)

ECOG = Eastern Cooperative Oncology Group, PS = Performance Status, NSCLC = Non Small Cell Lung Cancer, OMD = Oligometastatic disease; ^a^ Multiple organs with brain = lung + brain (8), adrenal + brain (6), bone + brain (2), liver + brain (2), spleen + brain (1), lymph node + brain (1), pancreas + kidney + brain (1); ^b^ Multiple organs extra brain = lung + liver (2), lung + adrenal (1), lung + kidney (1) + lung + liver + adrenal (1).

**Table 2:** Treatment characteristics.

Treatment Characteristics	All Patients, *n* = 245
	No. (%)
Treatment at NSCLC diagnosis	
Surgery alone	71 (29)
SABR	30 (12)
CT + RT	24 (10)
Systemic therapy alone	55 (22)
Surgery + adjuvant therapy	60 (25)
Other treatment	5 (2)
Systemic therapy at NSCLC diagnosis	
CT	129 (53)
IT	3 (1)
CT + IT	2 (1)
None	111 (45)
Response after NSCLC treatment	
CR	97 (40)
PR	91 (37)
SD	36 (15)
PD	21 (8)
Primary controlled at OMD diagnosis	
Yes	195 (80)
No	50 (20)
No. of treated lesion(s) with LAT	
1	187 (77)
2	48 (19)
3	9 (3.5)
4	1 (0.5)
No. of treated organ(s) with LAT at OMD diagnosis	
1	243 (99)
2	2 (1)
Cranial vs. Extracranial metastatic disease	
Cranial	128 (53)
Extracranial	117 (47)
Systemic treatment for metachronous OMD/OPD	
Yes	18 (7)
No	227 (93)
Additional non stereotactic local therapies	
Surgery	10 (4)
RFA	2 (1)
None	233 (95)
Adjuvant SRS/SABR	
Yes	8 (3)
No	237 (97)

NSCLC = Non Small Cell Lung Cancer, SABR = Stereotactic Ablative Body Radiotherapy, CT = Chemotherapy, RT = Radiotherapy, IT = Immunotherapy, CR = Complete Response, PR = Partial Response, SD = Stable Disease, PD = Progression Disease, OMD = Oligometastatic disease, OPD = Oligoprogressive Disease; RFA = Radiofrequency Ablation, SRS = Stereotactic Radiosurgery, LAT = local ablative therapy (SABR/SRS).

**Table 3:** Univariate and Multivariate Cox Regression of Factors Predicting Progression-Free Survival and Overall Survival.

Variable	Progression-Free Survival				Overall Survival			
	Univariate		Multivariate		Univariate		Multivariate	
	HR (95% CI)	*p* Value	HR (95% CI)	*p* Value	HR (95% CI)	*p* Value	HR (95% CI)	*p* Value
Age	0.98 (0.96–0.99)	0.009	NS	-	NS	-	NS	-
Stage IV at diagnosis	2.03 (1.47–2.81)	<0.001	NS	-	1.62 (1.14–2.30)	0.007	NS	-
Nodal involvement at baseline (N+)	1.70 (1.24–2.33)	0.001	NS	-	1.71 (1.20–2.45)	0.003	1.6 (1.1–2.4)	0.049
No response after primary treatment	2.04 (1.25–3.34)	0.005	NS	-	NS	-	NS	-
ECOG PS = 1	1.75 (1.27–2.41)	0.001	1.9 (1.3–2.6)	<0.001	2.06 (1.46–2.92)	<0.001	1.7 (1.1–2.6)	0.011
Type of oligometastatic disease	1.21 (1.07–1.37)	0.002	NS	-	NS	-	NS	-
No. of lesions at OMD diagnosis	1.27 (1.09–1.48)	0.003	NS	-	1.29 (1.07–1.55)	0.007	NS	-
Primary tumor uncontrolled	1.78 (1.24–2.55)	0.002	NS	-	1.65 (1.11–2.44)	0.013	NS	-
Intracranial metastatic disease	2.78 (2.00–3.83)	<0.001	2.4 (1.7–3.4)	<0.001	2.79 (1.90–4.10)	<0.001	2.3 (1.4–3.9)	0.001
CMT for OMD	2.5 (1.7–3.5)	<0.001	1.9 (1.3–2.6)	0.002	1.77 (1.21–2.57)	0.003	1.7 (1.1–2.6)	0.020
No response after LAT	4.36 (2.27–8.38)	<0.001	4.4 (2.2–8.5)	<0.001	NS	-	NS	-
New local treatment for PD	NS	-	NS	-	2.01 (1.35–3.00)	0.001	NS	-
No local treatment for PD vs. Intracranial vs. Extracranial new site of local treatment	NA	-	NA	-	1.85 (1.41–2.43)	<0.001	2.2 (1.6–3.1)	<0.001

ECOG = Eastern Cooperative Oncology Group, PS = Performance Status, OMD = oligometastatic disease, CMT = chemotherapy in combination with LAT, LAT = local ablative therapy (SABR/SRS), PD = progression disease, NS = not significant, NA = not available.

## Data Availability

All data of this study are stored at the Radiation Oncology Unit of the Oncology Department of the University of Torino.

## References

[1] Sung H., Ferlay J., Siegel R.L., Laversanne M., Soerjomataram I., Jemal A., Bray F. (2021). Global Cancer Statistics 2020: GLOBOCAN Estimates of Incidence and Mortality Worldwide for 36 Cancers in 185 Countries. CA Cancer J. Clin..

[2] Planchard D., Popat S., Kerr K., Novello S., Smit E.F., Faivre-Finn C., Mok T.S., Reck M., Van Schil P.E., Hellmann M.D. (2018). Metastatic Non-Small Cell Lung Cancer: ESMO Clinical Practice Guidelines for Diagnosis, Treatment and Follow-Up. Ann. Oncol..

[3] Hellman S., Weichselbaum R.R. (1995). Oligometastases. JCO.

[4] Lievens Y., Guckenberger M., Gomez D., Hoyer M., Iyengar P., Kindts I., Méndez Romero A., Nevens D., Palma D., Park C. (2020). Defining Oligometastatic Disease from a Radiation Oncology Perspective: An ESTRO-ASTRO Consensus Document. Radiother. Oncol..

[5] Guckenberger M., Lievens Y., Bouma A.B., Collette L., Dekker A., deSouza N.M., Dingemans A.-M.C., Fournier B., Hurkmans C., Lecouvet F.E. (2020). Characterisation and Classification of Oligometastatic Disease: A European Society for Radiotherapy and Oncology and European Organisation for Research and Treatment of Cancer Consensus Recommendation. Lancet Oncol..

[6] Lindeman N.I., Cagle P.T., Aisner D.L., Arcila M.E., Beasley M.B., Bernicker E.H., Colasacco C., Dacic S., Hirsch F.R., Kerr K. (2018). Updated Molecular Testing Guideline for the Selection of Lung Cancer Patients for Treatment With Targeted Tyrosine Kinase Inhibitors: Guideline From the College of American Pathologists, the International Association for the Study of Lung Cancer, and the Association for Molecular Pathology. Arch Pathol. Lab. Med..

[7] Maemondo M., Inoue A., Kobayashi K., Sugawara S., Oizumi S., Isobe H., Gemma A., Harada M., Yoshizawa H., Kinoshita I. (2010). Gefitinib or Chemotherapy for Non-Small-Cell Lung Cancer with Mutated EGFR. N. Engl. J. Med..

[8] Petrelli F., Ghidini A., Cabiddu M., Tomasello G., De Stefani A., Bruschieri L., Vitali E., Ghilardi M., Borgonovo K., Barni S. (2018). Addition of Radiotherapy to the Primary Tumour in Oligometastatic NSCLC: A Systematic Review and Meta-Analysis. Lung Cancer.

[9] Li X., Gomez D., Iyengar P. (2021). Local Ablative Therapy in Oligometastatic NSCLC. Semin Radiat. Oncol..

[10] Román-Jobacho A., Hernández-Miguel M., García-Anaya M.J., Gómez-Millán J., Medina-Carmona J.A., Otero-Romero A. (2021). Oligometastatic Non-Small Cell Lung Cancer: Current Management. J. Clin. Transl. Res..

[11] Mentink J.F., Paats M.S., Dumoulin D.W., Cornelissen R., Elbers J.B.W., Maat A.P.W.M., von der Thüsen J.H., Dingemans A.-M.C. (2021). Defining Oligometastatic Non-Small Cell Lung Cancer: Concept versus Biology, a Literature Review. Transl. Lung Cancer Res..

[12] Iyengar P., Wardak Z., Gerber D.E., Tumati V., Ahn C., Hughes R.S., Dowell J.E., Cheedella N., Nedzi L., Westover K.D. (2018). Consolidative Radiotherapy for Limited Metastatic Non-Small-Cell Lung Cancer: A Phase 2 Randomized Clinical Trial. JAMA Oncol..

[13] Gomez D.R., Tang C., Zhang J., Blumenschein G.R., Hernandez M., Lee J.J., Ye R., Palma D.A., Louie A.V., Camidge D.R. (2019). Local Consolidative Therapy Vs. Maintenance Therapy or Observation for Patients With Oligometastatic Non-Small-Cell Lung Cancer: Long-Term Results of a Multi-Institutional, Phase II, Randomized Study. J. Clin. Oncol..

[14] Palma D.A., Olson R., Harrow S., Gaede S., Louie A.V., Haasbeek C., Mulroy L., Lock M., Rodrigues G.B., Yaremko B.P. (2020). Stereotactic Ablative Radiotherapy for the Comprehensive Treatment of Oligometastatic Cancers: Long-Term Results of the SABR-COMET Phase II Randomized Trial. J. Clin. Oncol..

[15] Ko E.C., Raben D., Formenti S.C. (2018). The Integration of Radiotherapy with Immunotherapy for the Treatment of Non-Small Cell Lung Cancer. Clin. Cancer Res..

[16] Theelen W.S.M.E., Chen D., Verma V., Hobbs B.P., Peulen H.M.U., Aerts J.G.J.V., Bahce I., Niemeijer A.L.N., Chang J.Y., de Groot P.M. (2021). Pembrolizumab with or without Radiotherapy for Metastatic Non-Small-Cell Lung Cancer: A Pooled Analysis of Two Randomised Trials. Lancet Respir. Med..

[17] Ricardi U., Frezza G., Filippi A.R., Badellino S., Levis M., Navarria P., Salvi F., Marcenaro M., Trovò M., Guarneri A. (2014). Stereotactic Ablative Radiotherapy for Stage I Histologically Proven Non-Small Cell Lung Cancer: An Italian Multicenter Observational Study. Lung Cancer.

[18] Badellino S., Muzio J.D., Schivazappa G., Guarneri A., Ragona R., Bartoncini S., Trino E., Filippi A.R., Fonio P., Ricardi U. (2017). No Differences in Radiological Changes after 3D Conformal vs VMAT-Based Stereotactic Radiotherapy for Early Stage Non-Small Cell Lung Cancer. Br. J. Radiol..

[19] Filippi A.R., Badellino S., Guarneri A., Levis M., Botticella A., Mantovani C., Ragona R., Racca P., Buffoni L., Novello S. (2014). Outcomes of Single Fraction Stereotactic Ablative Radiotherapy for Lung Metastases. Technol. Cancer Res. Treat..

[20] Shaw E., Scott C., Souhami L., Dinapoli R., Kline R., Loeffler J., Farnan N. (2000). Single Dose Radiosurgical Treatment of Recurrent Previously Irradiated Primary Brain Tumors and Brain Metastases: Final Report of RTOG Protocol 90-05. Int. J. Radiat. Oncol. Biol. Phys..

[21] Mantovani C., Gastino A., Cerrato M., Badellino S., Ricardi U., Levis M. (2021). Modern Radiation Therapy for the Management of Brain Metastases From Non-Small Cell Lung Cancer: Current Approaches and Future Directions. Front Oncol..

[22] Benedict S.H., Yenice K.M., Followill D., Galvin J.M., Hinson W., Kavanagh B., Keall P., Lovelock M., Meeks S., Papiez L. (2010). Stereotactic Body Radiation Therapy: The Report of AAPM Task Group 101. Med. Phys..

[23] Hanna G.G., Murray L., Patel R., Jain S., Aitken K.L., Franks K.N., van As N., Tree A., Hatfield P., Harrow S. (2018). UK Consensus on Normal Tissue Dose Constraints for Stereotactic Radiotherapy. Clin. Oncol. (R. Coll. Radiol.).

[24] Eisenhauer E.A., Therasse P., Bogaerts J., Schwartz L.H., Sargent D., Ford R., Dancey J., Arbuck S., Gwyther S., Mooney M. (2009). New Response Evaluation Criteria in Solid Tumours: Revised RECIST Guideline (Version 1.1). Eur. J. Cancer.

[25] Ettinger D.S., Wood D.E., Aggarwal C., Aisner D.L., Akerley W., Bauman J.R., Bharat A., Bruno D.S., Chang J.Y., Chirieac L.R. (2019). NCCN Guidelines Insights: Non-Small Cell Lung Cancer, Version 1.2020. J. Natl. Compr. Canc. Netw..

[26] Kroeze S.G.C., Schaule J., Fritz C., Kaul D., Blanck O., Kahl K.H., Roeder F., Siva S., Verhoeff J.J.C., Adebahr S. (2021). Metastasis Directed Stereotactic Radiotherapy in NSCLC Patients Progressing under Targeted- or Immunotherapy: Efficacy and Safety Reporting from the “TOaSTT” Database. Radiat. Oncol..

[27] Ashworth A.B., Senan S., Palma D.A., Riquet M., Ahn Y.C., Ricardi U., Congedo M.T., Gomez D.R., Wright G.M., Melloni G. (2014). An Individual Patient Data Metaanalysis of Outcomes and Prognostic Factors after Treatment of Oligometastatic Non-Small-Cell Lung Cancer. Clin. Lung Cancer.

[28] Kissel M., Martel-Lafay I., Lequesne J., Faivre J.-C., Le Péchoux C., Stefan D., Barraux V., Loiseau C., Grellard J.-M., Danhier S. (2019). Stereotactic Ablative Radiotherapy and Systemic Treatments for Extracerebral Oligometastases, Oligorecurrence, Oligopersistence and Oligoprogression from Lung Cancer. BMC Cancer.

[29] Poon I., Erler D., Dagan R., Redmond K.J., Foote M., Badellino S., Biswas T., Louie A.V., Lee Y., Atenafu E.G. (2020). Evaluation of Definitive Stereotactic Body Radiotherapy and Outcomes in Adults With Extracranial Oligometastasis. JAMA Netw. Open.

[30] Buglione M., Jereczek-Fossa B.A., Bonù M.L., Franceschini D., Fodor A., Zanetti I.B., Gerardi M.A., Borghetti P., Tomasini D., Di Muzio N.G. (2020). Radiosurgery and Fractionated Stereotactic Radiotherapy in Oligometastatic/Oligoprogressive Non-Small Cell Lung Cancer Patients: Results of a Multi-Institutional Series of 198 Patients Treated with “Curative” Intent. Lung Cancer.

[31] Kim C., Hoang C.D., Kesarwala A.H., Schrump D.S., Guha U., Rajan A. (2017). Role of Local Ablative Therapy in Patients with Oligometastatic and Oligoprogressive Non-Small Cell Lung Cancer. J. Thorac. Oncol..

[32] Parikh R.B., Cronin A.M., Kozono D.E., Oxnard G.R., Mak R.H., Jackman D.M., Lo P.C., Baldini E.H., Johnson B.E., Chen A.B. (2014). Definitive Primary Therapy in Patients Presenting with Oligometastatic Non-Small Cell Lung Cancer. Int. J. Radiat. Oncol. Biol. Phys..

[33] De Ruysscher D., Wanders R., van Baardwijk A., Dingemans A.-M.C., Reymen B., Houben R., Bootsma G., Pitz C., van Eijsden L., Geraedts W. (2012). Radical Treatment of Non-Small-Cell Lung Cancer Patients with Synchronous Oligometastases: Long-Term Results of a Prospective Phase II Trial (Nct01282450). J. Thorac. Oncol..

[34] Li S., Zhu R., Li D., Li N., Zhu X. (2018). Prognostic Factors of Oligometastatic Non-Small Cell Lung Cancer: A Meta-Analysis. J. Thorac. Dis..

[35] Onishi H., Shirato H., Nagata Y., Hiraoka M., Fujino M., Gomi K., Niibe Y., Karasawa K., Hayakawa K., Takai Y. (2007). Hypofractionated Stereotactic Radiotherapy (HypoFXSRT) for Stage I Non-Small Cell Lung Cancer: Updated Results of 257 Patients in a Japanese Multi-Institutional Study. J. Thorac. Oncol..

[36] Lehrer E.J., Peterson J.L., Zaorsky N.G., Brown P.D., Sahgal A., Chiang V.L., Chao S.T., Sheehan J.P., Trifiletti D.M. (2019). Single versus Multifraction Stereotactic Radiosurgery for Large Brain Metastases: An International Meta-Analysis of 24 Trials. Int. J. Radiat. Oncol. Biol. Phys..

[37] Amini A., Verma V., Simone C.B., Chetty I.J., Chun S.G., Donington J., Edelman M.J., Higgins K.A., Kestin L.L., Movsas B. (2022). American Radium Society Appropriate Use Criteria for Radiation Therapy in Oligometastatic or Oligoprogressive Non-Small Cell Lung Cancer. Int. J. Radiat. Oncol. Biol. Phys..

[38] Basler L., Kroeze S.G.C., Guckenberger M. (2017). SBRT for Oligoprogressive Oncogene Addicted NSCLC. Lung Cancer.

